# Investments in Research and Surveillance Are Needed to Go Beyond Elimination and Stop Transmission of *Leishmania* in the Indian Subcontinent

**DOI:** 10.1371/journal.pntd.0005190

**Published:** 2017-01-26

**Authors:** Piero L. Olliaro, Tushar A. K. M. Shamsuzzaman, Baburam Marasini, A. C. Dhariwal, Ahmed Be-Nazir, Dinesh Mondal, Megha Raj Banjara, Pradeep Das, Shyam Sundar, Suman Rijal, Byron Arana, Jorge Alvar, Daniel Argaw, Rosanna W. Peeling, Axel Kroeger, Greg Matlashewski

**Affiliations:** 1 UNICEF/UNDP/World Bank/WHO Special Programme for Research & Training in Tropical Diseases (TDR), Geneva, Switzerland; 2 Centre for Tropical Medicine and Global Health, Nuffield Department of Medicine, University of Oxford, Oxford, United Kingdom; 3 Directorate General of Health Services (DGHS), Ministry of Health, Dhaka, Bangladesh; 4 Epidemiology and Disease Control Division, Department of Health Services, Ministry of Health, Kathmandu, Nepal; 5 National Vector Borne Disease Control Programme (NVBDCP), Directorate General of Health Services, Ministry of Health, New Delhi, India; 6 National Institute of Preventive and Social Medicine (NIPSOM), Ministry of Health, Dhaka, Bangladesh; 7 International Centre for Diarrhoeal Diseases Research, Bangladesh (ICDDRB), Dhaka, Bangladesh; 8 Central Department of Microbiology, Tribhuvan University, Kathmandu, Nepal; 9 Rajendra Memorial Research Institute (RMRI)–ICMR, Patna, India; 10 Department of Medicine, Banaras Hindu University, Varanasi, India; 11 Drugs for neglected Diseases initiative (DNDi) Regional Office, New Delhi, India; 12 Drugs for neglected Diseases initiative DNDi, Geneva, Switzerland; 13 Neglected Tropical Diseases (NTD) Department, World Health Organization, Geneva, Switzerland; 14 London School of Hygiene and Tropical Medicine, London, United Kingdom; 15 Centre for Medicine and Society, Department of Anthropology, University of Freiburg, Freiburg, Germany; 16 Department of Microbiology and Immunology, McGill University, Montreal, Canada; London School of Hygiene and Tropical Medicine, UNITED KINGDOM

Progress towards the elimination of a neglected tropical disease from a country can sometimes be a curse, especially when policymakers are confronted with competing needs and priorities. This could mistakenly be interpreted as though the disease has been eradicated, resulting in the limited resources becoming redirected to the next priority, sometimes leaving unfinished agendas. Much depends on whether the long term is considered in the elimination agenda and whether contingency plans are in place should the disease re-emerge as a public health problem.

This is the challenge countries in the Indian subcontinent are currently facing. In 2005, the governments of India, Nepal, and Bangladesh, in collaboration with World Health Organization (WHO) and later joined by Bhutan and Thailand, developed a strategic framework to eliminate visceral leishmaniasis as a public health problem (Kala-azar Elimination Programme). “Elimination” is one of the targets (ranging from “control” to “eradication”) of public health interventions against infectious diseases [1]. In the case of the Kala-azar Elimination Programme in the Indian subcontinent, achievement of elimination is quantified as reaching and maintaining a target of less than one case per 10,000 people in all endemic areas for three consecutive years at the district or sub-district level.

The programme has three phases: attack (bring down cases to below 1/10,000 by 2017), consolidation (case levels below 1/10,000 for three years, from 2017–2019), and maintenance (case levels below 1/10,000 beyond 2020) [2,3]. This seems within reach: 2015 was the third year in a row that Nepal has been consistently below that target [4, 5], and Bangladesh has only a few districts (“upazilas”) left that remain above the target [5]. India, which started with a higher prevalence of the disease than the other nations, has managed to significantly reduce levels in the majority of endemic districts.

Solid progress in the attack phase of the Kala-azar Elimination Programme has been possible largely through the availability of a point-of-care rapid diagnostic test (the rK39 RDT) and improved treatments (liposomal amphotericin B, paromomycin, and miltefosine, which have been variably tested and deployed alone or in combination in the three countries) [6] at primary health centers (PHC) close to the endemic communities and at referral hospitals. This has been coupled with improved vector control and increased knowledge in the communities that treatments are available and free.

However, this does not mean that the game is over. First, although this has resulted in decreases in morbidity and mortality, thousands of cases remain in the highly endemic areas in India. Moreover, considering the high population density in endemic states such as Bihar, there will continue to be thousands of new cases each year despite reaching the elimination target. Consequently, the possibility of major new outbreaks will remain high and could result in a resurgence of visceral leishmaniasis throughout the Indian subcontinent. Second, pockets of transmission remain in nonprogramme areas. Third, the approaches which have been used so far and proved successful in bringing high prevalence rates down or close to the elimination target may not be appropriate and sustainable in the consolidation and maintenance phase. Lastly, we do not know what the one-in-ten-thousand target really means in terms of transmission (we do not know how many new infections a case will generate [the “basic reproduction number” or R_0_, in epidemiological terms]).

Considering that humans are believed to be the only reservoir for transmission, it may therefore be time to reconsider a different target based on reduction in transmission. The objective should be to reduce incidence (rather than prevalence) by maintaining zero new cases in areas that have reached the elimination target. To maintain this target and stop transmission, it is necessary to re-evaluate whether the appropriate interventions are in place for surveillance, diagnosis, treatment, and vector control. In particular, it is important to decide whether current approaches should be reconsidered and whether new or modified interventions are required.

The current strategy in the Indian subcontinent is built around the early detection of symptomatic cases so that morbidity and mortality can be reduced and the source of infection is removed from the community. To identify these cases, the rK39 RDT, which detects anti-leishmania antibodies (i.e., the signature of infection, not the presence of parasites), is currently deployed as part of an algorithm whereby subjects with clinical symptoms suggestive of visceral leishmaniasis (fever lasting for at least two weeks and splenomegaly) are diagnosed as new cases and treated if they test positive on the rK39 RDT. The rK39 RDT is highly sensitive and specific [7] and, under these conditions, has been effective in the attack phase of the Kala-azar Elimination Programme.

However, currently we cannot distinguish a person with a past infection from someone with an active infection (no validated antigen-based RDT is available [7]), and it is not possible to quantify parasite load. A noninvasive, antigen-based RDT, similar to the ones available for malaria, to measure high parasite load would help reduce transmission. For example, identifying asymptomatic individuals with high parasite load could potentially identify individuals that can transmit the infection and become diseased [8,9]; these individuals and their family members and neighbors could be closely monitored for symptoms. A diagnostic test to measure parasite load is also needed as a test of cure to monitor the efficacy of current treatments and to improve the efficiency of research and development for new drugs. This cannot be done with the rK39 RDT, as it takes a long time for antibody levels to wane. An assay to measure parasite load could also be helpful in vaccine trials to monitor the efficacy of therapeutic vaccines or vaccination of individuals with asymptomatic infections. Although sensitive and specific nucleic acid amplification tests are potentially available for deployment in point-of-care format [10, 11, 12], these will require further validation and standardization in the field. Research is also needed to determine the threshold level of parasites associated with progression to disease and transmission to sandflies and whether an antigen-based test would be sensitive enough to meet this threshold.

As the number of visceral leishmaniasis cases continues to go down, however, fewer fever cases will result from *Leishmania* infections. To ensure that we will be able to continue to detect visceral leishmaniasis, the health system will have to evolve to have a better understanding of other febrile infections, including viral, bacterial, and parasitic infections.

Current treatments for visceral leishmaniasis are largely effective in the Indian subcontinent but do not completely eradicate the infection; they reduce the parasite burden to a level that can be controlled by a healthy immune system. Recrudescences occur in the presence of a deficient immune system, as is the case in HIV–*Leishmania* co-infected subjects [13]. There is, therefore, a need for sterilising drugs, possibly administered orally over a short period, since the only available oral drug (miltefosine) requires 28 days if used alone and may be teratogenic (and, therefore, should not be taken by women of childbearing age who are not using contraception).

Better treatments for PKDL are also a priority, since lesions can harbour parasites for years and, therefore, could be a reservoir to maintain the parasite in the community over long periods of time [8]. This could be one area where immunotherapy or a therapeutic vaccine may have merit, since current PKDL treatment regimens lasts for months (resulting in increased levels of adverse events) and are poorly adhered to [14].

Regardless of what treatments are available now and in the future, perhaps the most important consideration is to ensure that treatments and diagnostic tests are available at all of the PHCs in endemic communities. This will require a greater number of PHCs with sufficient expertise to deliver the first-line treatment, which is currently single-dose liposomal amphotericin B. Access to treatment locally, combined with increased knowledge in the community, will reduce the time from symptoms to treatment and, consequently, reduce the rate of transmission. This can be achieved immediately with the necessary commitment.

Finally, a vaccine to stop transmission would represent the best intervention [15]. People who are cured of visceral leishmaniasis following treatment are immune against re-infection for life, which implies that a vaccine for leishmaniasis is possible. Due to the relatively low numbers of cases and high population, it would be necessary in the first instance to combine a vaccination programme with the elimination programme to deploy the vaccine in highly endemic areas and areas with new outbreaks during the maintenance phase. Any vaccine would need to be effective on both immunologically naive people and asymptomatic infected people if it is to be deployed in highly endemic areas.

The important achievements of the country programmes in the Indian subcontinent over the past ten years deserve high praise. Additional attention from endemic country governments and investments by the international community are now required so that recent advancements are sustainable, visceral leishmaniasis can sustainably be eliminated, and transmission reaches zero. This situation is not new, nor is it unique to visceral leishmaniasis. The case of the leprosy elimination programme, which set the same one case per 10,000 population target, is a lesson that elimination targets must be contextualised and does not mean that when the target is reached the disease is gone (eradicated) [16].

A recent paper from an authoritative group points to the need for translational science and new technologies, and that elimination requires new tools for a range of neglected tropical diseases [17]. Gaps in diagnosis, treatment, vector control, and vaccines, some of which are identified above, must be addressed through continuing support for research for innovation that will help close the gaps. In the meantime, it is imperative that, through education, surveillance, and operational research, the *Leishmania*-exposed populations in the villages become the focus of the response with the tools available to block transmission and stop micro-outbreaks from spreading. Continued investment in research aimed at reaching the goal of stopping visceral leishmaniasis transmission would ensure that the tremendous investments by governments, donors, and the international community to date have not been in vain.
